# Frequency of data extraction errors and methods to increase data extraction quality: a methodological review

**DOI:** 10.1186/s12874-017-0431-4

**Published:** 2017-11-28

**Authors:** Tim Mathes, Pauline Klaßen, Dawid Pieper

**Affiliations:** Institute for Research in Operative Medicinem, Chair of Surgical Research, Faculty of Health, School of Medicine, Witten/Herdecke University, Ostmerheimer Str. 200, 51109 Cologne, Germany

**Keywords:** Systematic reviews, Data extraction, Accuracy, Errors, Reviewers

## Abstract

**Background:**

Our objective was to assess the frequency of data extraction errors and its potential impact on results in systematic reviews. Furthermore, we evaluated the effect of different extraction methods, reviewer characteristics and reviewer training on error rates and results.

**Methods:**

We performed a systematic review of methodological literature in PubMed, Cochrane methodological registry, and by manual searches (12/2016). Studies were selected by two reviewers independently. Data were extracted in standardized tables by one reviewer and verified by a second.

**Results:**

The analysis included six studies; four studies on extraction error frequency, one study comparing different reviewer extraction methods and two studies comparing different reviewer characteristics. We did not find a study on reviewer training. There was a high rate of extraction errors (up to 50%). Errors often had an influence on effect estimates. Different data extraction methods and reviewer characteristics had moderate effect on extraction error rates and effect estimates.

**Conclusion:**

The evidence base for established standards of data extraction seems weak despite the high prevalence of extraction errors. More comparative studies are needed to get deeper insights into the influence of different extraction methods.

**Electronic supplementary material:**

The online version of this article (10.1186/s12874-017-0431-4) contains supplementary material, which is available to authorized users.

## Background

Systematic reviews (SRs) have become the cornerstone of evidence based healthcare. A SR should use explicit methods to minimize bias with the aim to provide more reliable findings [1]. The reduction of bias concerns all process steps of the review. For example, bias can occur in the identification of studies, in the selection of studies (e.g. unclear inclusion criteria), in the data collection process and in the validity assessment of included studies [2]. Many efforts have been made to further develop methods for SRs.

However, the evidence base for most recommendations that aim to minimize bias in the preparation process for a systematic review in established guidelines is sparse [1, 3, 4]. Previous studies found only little research on the influence of different approaches on risk of bias in systematic reviews [5]. The use of work extensive methods without really knowing the benefit might waste scientific resources. For example a recent study found that alternatives to duplicate study selection by two independent reviewers (e.g. liberal acceleration) are more cost-effective than independent study selection [6]. As the timely preparation and publication of SR is an important goal to support decision making considerations on the balance between resources/time and validity play an important role [7].

Data extraction is a crucial step in conducting SRs. The term *data collection* is often used synonymously. We defined data extraction as any type of extracting data from primary studies into any form of standardized tables. It is one of the most time-consuming and most critical tasks for the validity of results of a SR [1]. Data extraction builds the basis for the results and conclusion in a SR. However, a previous study has shown a very high prevalence of data extraction errors in SRs [8].

Our objective was to assess the frequency of data extraction errors and its potential impact on results in systematic reviews of interventions. Furthermore, we evaluated the effect of different extraction methods (e.g. independent data extraction by two reviewers vs. verification by a second reviewer), reviewer characteristics (e.g. experience) and reviewer training on error rates and results.

## Methods

### Information sources and search

We searched all PubMed databases and the Cochrane Methodology Register (12/2016). The full search strategies are provided in Additional file 1. The search strategies were tested by checking whether articles already known to us (e.g. Buscemi et al. [9]) were identified. We screened all abstracts of the Cochrane Colloquium (since 2009) and searched the Cochrane database of oral, poster and workshop presentations (since 1994) in December 2016. In addition we cross checked the references-list of all included articles, excluded articles, established guidelines for systematic review preparation and systematic reviews on similar topics [1, 3–5, 7, 10]. Moreover, we screened the publications linked in the related articles functions (cited articles and similar articles) in PubMed for all included articles.

### Eligibility criteria and study selection

Two types of articles were eligible. First, we included articles on the frequency of data extraction errors. Second, we included studies that compared aspects that can influence the quality of data extraction. We considered the following types of comparisons:

- Comparison of different methods (index methods) for data extraction regarding the involvement of a second reviewer for quality assurance (e.g. independent data extraction versus extraction by one reviewer and verification by a second) for data extraction (in the following called extraction method),
- Reviewer characteristics: experience, degree, education,
- Reviewer training: training on data extraction before performing the review, including extraction of a sample and calibration with the second reviewer and oral and written instructions.

Studies on technical tools (software, dual monitors, data extraction templates etc.) to reduce data extraction errors were excluded.

All studies (studies on error frequency and comparative studies) had to report a quantitative measure for data extraction errors (e.g. error rate), determined by use of a reference standard (a data extraction sample that was considered correct). We included only studies on the assessment of data extraction for intervention reviews written in English or German.

All titles/abstracts identified in the electronics databases were screened by two reviewers independently. The abstracts of the Cochrane Colloquium and the database of oral poster and workshop presentations were screened by one reviewer. All potentially relevant full-texts were screened by two reviewers (not independently). In case of discrepancies, we discussed eligibility until consensus.

### Data collection and analysis

We used a semi-structured template for data extraction (see Tables 1, 2 and 3). For each study, we extracted the sample size (the number of included studies and, if applicable the number of systematic reviews feeding the sample), information on the index and reference method, and information on outcome measures. We extracted all data on data extraction errors and the influence on pooled effect estimates. If possible, we distinguished data extraction errors in accuracy (e.g. correct values), completeness (e.g. all relevant outcomes are extracted)/selection (e.g. choice of correct outcome measure or outcome measurement time point) and correct interpretation (e.g. confuse mean and median). In addition, we extracted quantitative measures for effort (e.g. time, resource use) and rates of agreement between different approaches.

**Table 1:** Results of studies quantifying the frequency of data extraction errors

Study	Studies included (reviews)	Measure	Result
*Carroll 2013* [16]	8 (3)	Selection (outcome 1)	17% (review 1 vs. reference standard); 8% (review 2 vs. reference standard)
		Selection (outcome 2)	42% (review 1 vs. reference standard); 25% (review 2 vs. reference standard)
		Selection (outcome 3)	21% (review 1 vs. reference standard); 25% (review 2 vs. reference standard)
		Inaccuracy (outcome 1)	8% (review 1 vs. reference standard); 8% (review 2 vs. reference standard)
		Inaccuracy (outcome 2)	17% (review 1 vs. reference standard); 13% (review 2 vs. reference standard)
		Inaccuracy (outcome 3)	13% (review 1 vs. reference standard); 8% (review 2 vs. reference standard)
		Difference in meta-analysis (outcome 1)	RR 1.70 (reference standard) / RR 1.71 (review 1)
		Difference in meta-analysis (outcome 2)	RR 0.85 (reference standard) / RR 0.87 (review 1) / RR 0.80 (review 2)
		Difference in meta-analysis (outcome 3)	RR 0.38 (reference standard) / RR 0.40 (review 1)
*Gøtzsche 2007* [8]	54 (random selected; 27 meta-analysis)	Difference in SMD >0.1 of at least 1 of the 2 included trials	63%
	20 (10 meta-analysis^a^)	Difference in SMD >0.1 of pooled effect estimate.	70%
*Jones 2005* [17]	NR (34)	Errors (all types)	50%
		Correct interpretation	23.3%
		Impact on results	All data-handling errors led to changes in the summary results, but none of them affected review conclusions^b^
*Tendal 2009* [15]	45 (10 meta-analysis)	Difference in SMD because of reviewer disagreements < 0.1	53%
		Difference in SMD because of reviewer disagreements < 0.1 (pooled estimates)	31%

^a^: meta-analyses at least including one erroneous trial; ^b^author statement (no quantitative measures provided); *ns* no significant differences; *NR* not reported, *RD* relative difference, *RS* reference standard, *SMD* standardized mean difference

**Table 2:** Characteristics of studies comparing different reviewer extraction methods and reviewer characteristics

Study	Comparator/s		Reference^a^	Studies included
*Buscemi 2006* [9]	One reviewer verification by a second	Two reviewers independently	Extraction by one reviewer and verification by an experienced statistician	*N* = 30 (6 meta-analysis)
*Horton 2010* [14]	Minimal data extraction experience (*n* = 28)	Moderate data extraction experience (*n* = 19)	Substantial data extraction experience (*n* = 23)	NA
	Minimal systematic review experience (*n* = 28)	Moderate systematic review experience (*n* = 31)	Substantial systematic review experience (*n* = 18)	NA
	Minimal overall experience^b^ (*n* = 26)	Moderate overall experience^b^ (*n* = 24)	Substantial overall experience^b^ (*n* = 37)	NA
*Tendal 2009* [15]	Experienced methodologists	PhD students	No reference standard (comparison of raw agreement between reviewers)	45 (10 meta-analysis)

^a^denominator or subtrahend; ^b^based on time involved in systematic reviews and data extraction and the number of systematic reviews; *NA* not applicable

**Table 3:** Results of studies comparing different reviewer extraction methods and reviewer characteristics

	Measure	Result (effect measure, CI or *p*-value)
Reviewer constellation		
*Buscemi 2006* [9]	Agreement rate	28.0% (95% CI: 25.4, 30.7, range 11.1–47.2%)
	Errors (all types)	RD 21.7% (*p* = 0.019)
	Omission	RD 6.6% (*p* = 0.308)
	Time (min, mean)	RD −49 (*p* = 0.03)
	Difference of pooled effect estimates *	0/0
*Reviewer experience*		
*Horton 2010* [14]	Errors (all types)	24.3%/26.4%/25.4% (*p* = 0.91)
	Inaccuracy	14.3%/13.6%/15.7% (*p* = 0.41)
	Omission	10.0%/12.1%/12.1% (*p* = 0.24)
	Time (min, mean)	200/149/163 (p = 0.03)
	MD in point estimates of meta -analysis	ns (5 outcomes)
	Errors (all types)	25.0%/ 26.1%/ 24.3% (*p* = 0.73)
	Inaccuracy	14.6%/ 13.2%/ 15.7% (*p* = 0.39)
	Omission	10.0%/ 11.4%/ 10.7% (*p* = 0.53)
	Time (min, mean)	198/179/ 152 (*p* = 0.01)
	MD in point estimates of meta -analysis	ns (5 outcomes)
	Errors (all types)	26.4%/27.9%/27.9% (*p* = 0.73)
	Inaccuracy	16.4%/12.1%/15.7% (*p* = 0.22)
	Omission	10.4%/12.1%/13.6% (*p* = 0.47)
	Time (min, mean)	211/180/173 (*p* = 0.12)
	MD in point estimates of meta -analysis	ns (5 outcomes)
*Tendal 2009* [15]	Difference of SMD < 0.1	61%/46% (NR)
	Difference of SMD < 0.1 (pooled estimates)	33%/27% (NR)

*p < 0.05; *CI* confidence interval, *MD* mean difference, *ns* not statistical significant differences (according to authors, significance not specified), *NR* not reported, *RD* relative difference, *SMD* standardized mean difference

If provided in the article, we extracted confidence limits (preferred) or *p*-values for all outcomes/comparisons. Rates with a denominator of at least 10 were converted into percentages to increase the comparability of the results.

All data were extracted in standardized tables. Before starting the data extraction the involved reviewers discussed each article to agree about the relevant items for data extraction to avoid misinterpretation and omission. Subsequently, one reviewer (experienced statistician) extracted all data and a second reviewer (experienced epidemiologist) verified the accuracy of data extraction.

### Synthesis of data

Each included article was summarized in a structured narrative way. The narrative synthesis includes information on the sample (included reviews, included studies), the index method, the reference standard considered as correct data extraction and results (measures for the quantification of errors and measures for the quantification of influence on the effect estimates).

## Results

### Study selection

**Fig. 1:**
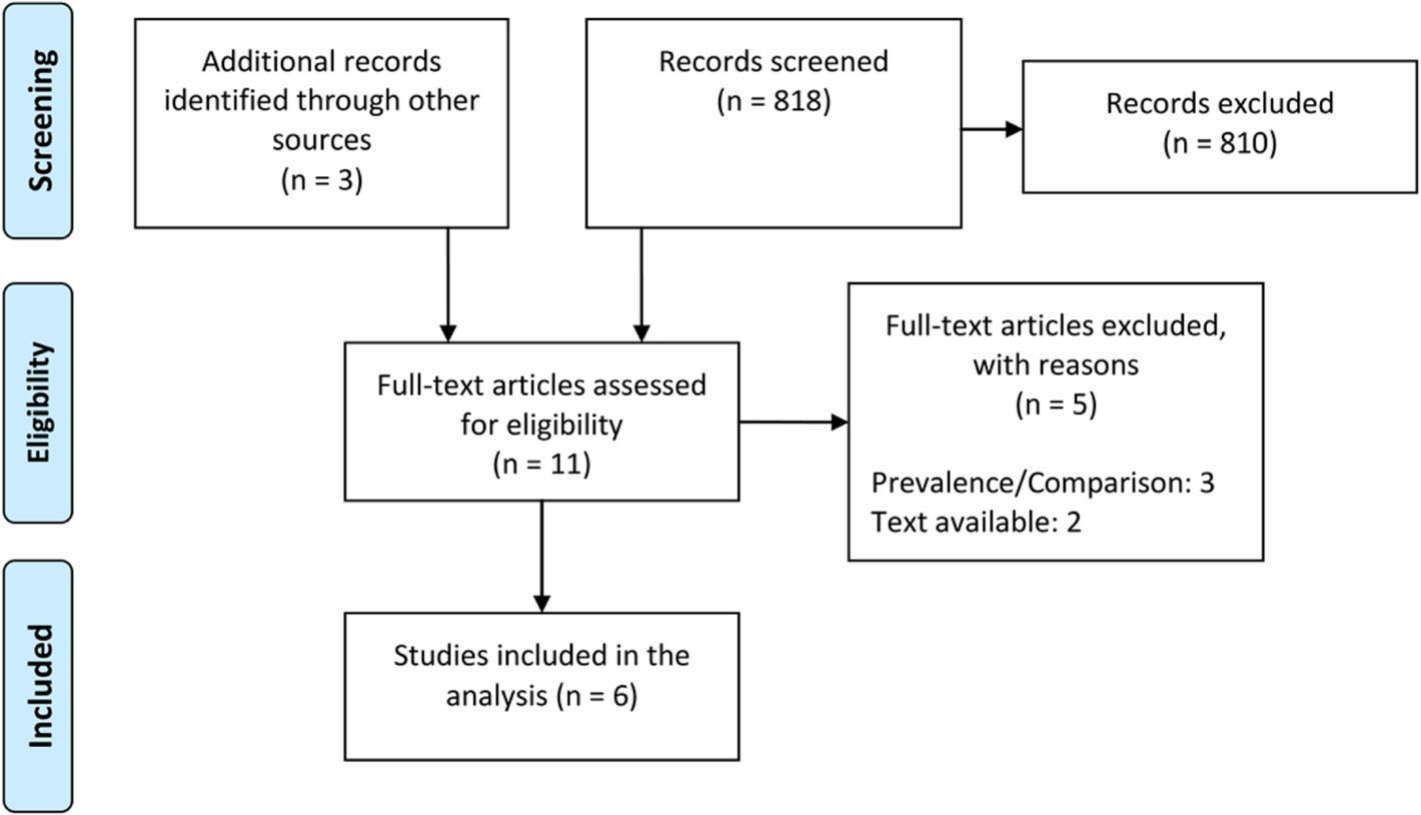
Flow-diagram of study selection

The search in the electronic databases resulted in 818 hits. The search of the abstracts of the Cochrane Colloquium and the database of the Cochrane database of oral, poster and workshop presentations revealed three additional potentially relevant publications. Additionally, the reference list of included articles and systematic reviews on similar topics did not reveal further relevant articles. For two studies, no full-texts were available. We screened the full-texts of nine articles. Of these, three studies were excluded [11–13]. The study selection process is illustrated in the flow-diagram (Fig. 1).

The analysis included six studies [8, 9, 14–17]; four studies on extraction error frequency [8, 15–17], one study comparing different reviewer extraction methods [9] and two studies comparing different reviewer characteristics [14, 15] (Tendal et al. [15] included in both analysis). No studies on reviewer training were identified.

### Studies on frequency of extraction errors

Carroll et al. compared the results of three dichotomous outcomes [16]. The database was three systematic reviews on the same topic that included the same studies (*N* = 8). Their own systematic review was used as the reference standard. Deviations in the other systematic reviews were considered as errors. The rate of data extraction errors ranged between 8% and 42% (depending on outcome and review). Differences in pooled effect estimates were small (difference of relative risk, range: 0.01–0.05).

Gøtzsche et al. [8] replicated the results of 27 meta-analyses. For the quantification of errors two trials of each meta-analysis were randomly selected. The reference standard was data extraction by two independent reviewers. A difference in the standardized mean difference of greater 0.1 was classified as error. In 63% of meta-analysis at least one of the two trials were erroneous. Of these meta-analyses 70% also showed an error of the pooled effect estimate.

In the study of Jones et al. [17] 42 systematic reviews of a Cochrane group were included. A statistician checked the data of these systematic reviews for errors. Half of the systematic reviews contained errors and approximately 23% of this were misinterpretations (e.g. confusing standard deviations and standard error). According to the study authors “all data-handling errors led to changes in the summary results, but none of them affected review conclusions” [17].

Tendal et al. [15] estimated the influence of deviations in data extractions between reviewers on results. A standardized mean difference smaller than 0.1 was considered as reviewer agreement. Approximately, 53% and 31% reviewers agreed on the trial level and meta-analysis level, respectively. At the level of meta-analysis the difference in standardized mean difference (SMD) was at least 1 in one of ten meta-analyses.

Table 1 shows the frequency of data extraction errors and influence on the effect estimates for each included study.

### Studies comparing different reviewer extraction methods and reviewer characteristics

Buscemi et al. [18] compared data extraction of 30 randomized controlled trials by two independent reviewers with data extraction by one reviewer and verification by a second. The reference standard was the data extraction by one reviewer with verification by an experienced statistician. The agreement rate of the two extraction methods was 28%. The risk difference of the total error rate was statistically significant (risk difference 21.7%, *p* = 0.019) in favor of double data extraction. This difference was primarily because of inaccuracy (risk difference 52.9%, *p* = 0.308). However, in average double data extraction took 49 min longer. Pooled effect estimates of both extraction methods varied only slightly and were not statistically significant different from the reference standard in any of the meta-analyses (*N* = 6).

In the study by Horton et al. [14] the data extractions of minimally experienced, moderately experienced and substantially experienced reviewers were compared. Experience classifications were based on years involved in systematic review preparation (≤2, 4–6, >7) and the number of performed data extractions (≤50, 51–300, >300). Three outcome measures were used; systematic review experience, extraction experience and a composite of these two (overall experience). The reference data extractions were prepared by independent data extraction by two reviewers and additional verification with the data in the original review. Error rates were similar across all overall experience levels. The median error rates ranged between 24.3%–26.4%, 13.6%–15.7% and 10.0%–12.1% for total errors, inaccuracy and omission, respectively (*p*-values >0.2 for all comparisons). Unexperienced reviewers required more time for data extraction (range: 173–211). There were no statistically significant differences (according authors, p-values not specified) in point estimates (5 outcomes) of meta-analysis between overall reviewer experience levels.

Tendal et al. [15] estimated the influence of deviations in data extractions between reviewers on results. Agreement was defined as a standardized mean difference for the effect estimates smaller than 0.1. Agreement was calculated for all pairs of experienced methodologist and all pairs of PhD students. Experienced methodologists agreed more than the PhD students (61% vs. 46% of trials; 33% vs. 27% of meta-analyses).

Table 2 shows the characteristics, and Table 3 the results of the comparison of different extraction methods and reviewer characteristics.

## Discussion

There is a high prevalence of extraction errors [8, 13, 15, 16]. However, extraction errors seem to have only a moderate impact on the results/conclusion of systematic reviews. Nevertheless, the high rate of extraction errors indicates that measures for quality assurance of data extraction are important to minimize the risk of biased results and wrong conclusions [8].

Comparative evidence on the influence of different reviewer extraction methods and reviewer characteristics is sparse [9, 14, 15]. Reviewer characteristics seem to have only a moderate influence on extraction errors [7, 9]. Data extraction by two independent reviewers seems to result in less extraction errors than data extraction by one reviewer and verification by a second. These large differences might cause significant difference in effect estimates [15]. However, in view of the limited influence on the conclusions of a systematic review, double data extraction of all data by two independent reviewers seems not always necessary, but reduced data extraction methods might be justified. The basic principal of reduced extraction methods is the focus on critical aspects (e.g. primary outcomes), which constitute the basis for conclusions, and reduced emphasize on the data extraction of less important aspects (e.g. patient characteristics, additional outcomes). Also in the Methodological Expectations of Cochrane Intervention Reviews (MECIR), it is stated that “dual data extraction may be less important for study characteristics than it is for outcome data, so it is not a mandatory standard for study characteristics” and “dual data extraction is particularly important for outcome data, which feed directly into syntheses of the evidence, and hence to the conclusions of the review” [19]. This is comparable to the recent policy of the Institute of Medicine (IOM). The IOM states that “at minimum, use two or more researchers, working independently, to extract quantitative and other critical data from each study” [20].

Such methods would reduce the effort for data extraction. Considering that the reviewer experience showed only little influence on extraction errors, cost might be further reduced by not employing only experienced methodologists but also staff with less systematic review experience for data extraction [14, 15]. However, reviewer training seems especially important, if less experienced reviewers are involved. The reviewer team should be trained in data extraction (e.g. using a sample) before performing the complete data extraction to harmonize data extraction and clear up common misunderstandings. This could in particular reduce interpretation and selection errors [13]. The reduction of time and effort is especially relevant for rapid reviews because this form of evidence synthesis aims timely preparation while remaining systematic [21]. Thus, clarifying which methods can reduce the time required for preparation without significantly increasing the risk of bias would also contribute to better control the short cuts in rapid reviews. The risk of bias of reduced data extraction methods could be further reduced if a detailed systematic review protocol and data entry instructions are prepared beforehand because the risk of selecting wrong outcomes (e.g. time points, measures) and omission would be reduced [15, 22].

The wide range of agreement between different extraction methods suggests that some studies are more difficult to extract than others [9]. There can be many reasons for this. First, data extraction is probably dependent on the reporting quality in primary studies. Often methods and results of trials are insufficiently reported [23, 24]. Bad reporting can complicate the identification of relevant information (e.g. primary outcome is not clearly indicated) and impede the extraction of treatment effects in a useful manner (e.g. only statements on statistical significance without effect measures and confidence intervals). Thus, bad reporting can increase the risk of omission and varying interpretation.

Second, the level of necessary statistical expertise might vary by study. Also reviewers who are familiar with a variety of statistical methods may not be aware of more advanced statistical methods (e.g. hierarchical regression models) or recently developed methods (e.g. mean cumulative function). Data extraction is particularly challenging when very different statistical methods (which can result in different effect estimates) are used across articles.

Third, different backgrounds (e.g. clinicians, epidemiologists) and levels of expertise might also play a role. However, drawing conclusions for practice seems difficult without an approach to differentiate between “easy” and “difficult” studies beforehand (e.g. tools to classify statistical complexity). Furthermore, it should be acknowledged that the complexity of data extraction not only depends on the data items in included studies but also on its aim. For example, to support data extraction a guide for complex meta-analysis (DECiMAL) has been recently published [10].

Findings are consistent across our included studies. Moreover, for short cuts in other process steps (e.g. quality assessment) that can have influence on the risk of bias in systematic reviews, similar results have been observed. For example, a study on the influence of searching multiple databases found only a weak impact of reducing the number of searched databases (because of not searching additional databases) on the results of meta-analysis and review conclusions [18, 25]. Therefore, also for other process steps, research on extraction methods seems to be needed. Future research should consider the influence on the risk of bias as well as the impact on scientific resources.

### Limitations

Although the included studies are probably not free from bias we did not perform a formal risk of bias assessment using a checklist because there is no established risk of bias tool for methodological studies. A source for risk of bias (correct extraction) is probably the imperfect reference standard in the included studies. For example, in the study by Jones et al. [17] only one statistician performed data extraction of results. Carroll et al. [16] considered their own systematic review as the reference standard. However, a perfect gold standard might be hardly achievable in such studies. Moreover, most studies considered only a certain research question regarding patients, intervention and comparison. The generalizability of the results is therefore unclear.

The search terms describing our research question were very unspecific (e.g. data collection). Because of this reason we used field limitations to balance the specificity and sensitivity. Therefore, we might not have identified all relevant articles.

We did not investigate novel computer-aided approaches for data extraction. Computer-aided data extraction can result in more efficiency and accuracy [26]. Although such approaches are only in their infancy, it can be expected that they will become more common in the future. Biomedical natural language processing techniques will be developed further in the near future [27].

## Conclusion

There is a high prevalence of extraction errors. This might cause relevant bias in effect estimates [8, 15–17]. However, there are only a few studies on the influence of different data extraction methods, reviewer characteristics and reviewer training on data extraction quality. Thus, the evidence base for the established standards of data extraction seems sparse because the actual benefit of a certain extraction method (e.g. independent data extraction) or the composition of extraction team (e.g. experience) is not sufficiently proven. This is surprising given that data extraction is a very crucial step in conducting a systematic review. More comparative studies are needed to get deeper insights into the influence of different extraction methods. In particular, studies investigating training for data extraction are needed because there is no such analysis, to date. Similar studies were recently published for risk of bias assessment [28]. The application of methods that require less effort without threating the internal validity would result in a more efficient utilization of scientific manpower. Increasing the knowledge base would also help to design effective training strategies for new reviewers and students in the future.

## Additional file

Additional file 1: Search strategies. (DOCX 13 kb)

## Abbreviations

SR: Systematic review
