# Supplementary material for: Frequency of data extraction errors and methods to increase data extraction quality: a methodological review
Source: BMC Med Res Methodol. 2017 Nov 28;17:152. doi: 10.1186/s12874-017-0431-4 (PMC5704562; doi:10.1186/s12874-017-0431-4)
Supplement: Additional file 1: — Search strategies. (DOCX 13 kb) [file 12874_2017_431_MOESM1_ESM.docx]

Additional file 1: Search strategies.

| **PubMed** | sysrev_methods [sb] AND ("reporting error" OR "observer agreement" OR "data extraction" OR "data collection" OR "data abstraction" OR "extraction error") |
| --- | --- |
| **Cochrane Methodology Register** | ("data extraction" OR "data collection" OR “extraction error"):ti |
